# Whole genome sequencing-based detection of antimicrobial resistance and virulence in non-typhoidal *Salmonella enterica* isolated from wildlife

**DOI:** 10.1186/s13099-017-0213-x

**Published:** 2017-11-21

**Authors:** Milton Thomas, Gavin John Fenske, Linto Antony, Sudeep Ghimire, Ronald Welsh, Akhilesh Ramachandran, Joy Scaria

**Affiliations:** 1 Department of Veterinary and Biomedical Sciences, South Dakota State University, Brookings, SD 57007 USA; 2 South Dakota Center for Biologics Research and Commercialization, Brookings, SD 57007 USA; 3 Oklahoma Animal Disease Diagnostic Laboratory, Oklahoma State University, Stillwater, OK 74078 USA

**Keywords:** Wildlife, Salmonellosis, Whole genome sequencing, Antimicrobial resistance, Salmonella virulence, Foodborne pathogen

## Abstract

The aim of this study was to generate a reference set of *Salmonella enterica* genomes isolated from wildlife from the United States and to determine the antimicrobial resistance and virulence gene profile of the isolates from the genome sequence data. We sequenced the whole genomes of 103 *Salmonella* isolates sampled between 1988 and 2003 from wildlife and exotic pet cases that were submitted to the Oklahoma Animal Disease Diagnostic Laboratory, Stillwater, Oklahoma. Among 103 isolates, 50.48% were from wild birds, 0.9% was from fish, 24.27% each were from reptiles and mammals. 50.48% isolates showed resistance to at least one antibiotic. Resistance against the aminoglycoside streptomycin was most common while 9 isolates were found to be multi-drug resistant having resistance against more than three antibiotics. Determination of virulence gene profile revealed that the genes belonging to *csg* operons, the *fim* genes that encode for type 1 fimbriae and the genes belonging to type III secretion system were predominant among the isolates. The universal presence of fimbrial genes and the genes encoded by pathogenicity islands 1–2 among the isolates we report here indicates that these isolates could potentially cause disease in humans. Therefore, the genomes we report here could be a valuable reference point for future traceback investigations when wildlife is considered to be the potential source of human Salmonellosis.

**Electronic supplementary material:**

The online version of this article (10.1186/s13099-017-0213-x) contains supplementary material, which is available to authorized users.

## Background

*Salmonella enterica* is the leading cause of foodborne illness in the United States accounting for approximately 1.2 million infections, 23,000 hospitalizations and 450 deaths annually. Over the past few decades, *Salmonella* has acquired new virulence determinants that influence host-tropism which helps these organisms to adapt to a wide range of hosts [1]. Multiple serovars of *S*. *enterica* originating from mammalian, reptilian and avian hosts have been reported to cause infections in humans [1]. Wildlife and exotic pets harboring *Salmonella* are potential sources for human infections [1]. Transmission of *Salmonella* from wildlife and exotic animals to humans occurs through multiple pathways. Increasing evidence suggests that there could be a bidirectional transmission of *Salmonella* between domesticated and wild animals. Farm animals acquiring *Salmonella* from wildlife could increase the risk of human infection. *Salmonella* infections in humans have also been reported through direct contact with exotic pets and wildlife, especially those in captivity. Consumption of contaminated game bird meat is also a potential source for foodborne salmonellosis. Furthermore, wildlife such as rodents and birds, harboring in the proximity of food production units can act as carriers and contaminate food products leading to indirect infections.

The threat posed by salmonellosis is further compounded by the presence of resistance genes that confer resistance to multiple antimicrobial drugs. According to the National Antimicrobial Resistance Monitoring System (NARMS) integrated report, 20% of human *Salmonella* isolates exhibit antimicrobial resistance (AMR). Antimicrobial-resistant *Salmonella* infections result in increased disease severity and longer hospitalizations in addition to economic losses [2]. Research indicates that *Salmonella* isolates from various wildlife species also possess AMR determinants and the prevalence rate of AMR genes in these isolates could be as high as 100% [3, 4]. Thus, *Salmonella* in wildlife poses a significant risk to human health underlining the need for an integrative ‘One Health’ approach for the surveillance of pathogens among humans, domestic animals, and wildlife population.

Whole genome sequencing (WGS) of foodborne pathogens could be adopted as an effective and rapid surveillance tool. Compared to conventional antimicrobial tests, WGS offers a more comprehensive information on the genotypic characteristics of pathogens including identification of AMR and virulence determinants, and serotypes. Recent studies have utilized WGS to reliably predict the antimicrobial characteristics in various pathogens including *Salmonella* [5–8]. In this study, WGS was utilized to predict AMR and virulence determinants in *Salmonella* isolated from exotic pets and wildlife.

## Methods

### Quality assurance

All strains were identified as *Salmonella enterica* following the American Association of Veterinary Laboratory Diagnosticians certified laboratory. For genome sequencing, each isolate was streaked on Salmonella selective medium and a single colony was picked and used for further steps as outlined below.

### Salmonella bacterial isolates

A total of 103 *Salmonella* isolates were revived from archival cultures obtained from exotic pet or wildlife clinical specimens submitted to the Oklahoma Animal Disease Diagnostic Laboratory, Stillwater, Oklahoma during 1988–2003. The metadata for the samples used in this study is provided in Table 1 and the details of genome sequencing and assembly parameters are given in Additional file 1: Table S1. Isolates were streaked on Luria–Bertani agar slants and were transported to the Animal Disease Research and Diagnostic Laboratory, South Dakota State University, Brookings, South Dakota for WGS. Samples were streaked on Luria–Bertani agar plates upon arrival to the laboratory. A single bacterial colony from the agar plate was then inoculated to Luria–Bertani broth and cultured at 37 °C.

**Table 1:** List of *Salmonella enterica* strains isolated and sequenced from wild life and the corresponding metadata

Strain ID	Serovar	Year	Animal	NCBI SRA BioSample ID	NCBI SRA ID
ADRDL-001	Poona	1993	Alligator omentum	SAMN06330630	SRR5278825
ADRDL-002	Typhimurium	1993	Auodad feces	SAMN06330629	SRR5278822
ADRDL-003	Gaminara	1994	Ratite intestine	SAMN06330628	SRR5278823
ADRDL-004	Lille	1993	Gamebird embryo	SAMN06330627	SRR5278827
ADRDL-005	Typhimurium	1993	Ratite feces	SAMN06333495	SRR5278819
ADRDL-006	Typhimurium	1993	Ratite feces	SAMN06333494	SRR5278824
ADRDL-007	Thompson	1993	Ratite cecum	SAMN06333493	SRR5278802
ADRDL-008	Livington	1993	Ratite cecum	SAMN06333492	SRR5278806
ADRDL-009	Typhimurium	1993	Ratite feces	SAMN06333491	SRR5278801
ADRDL-010	Montevideo	1993	Ratite feces	SAMN06333489	SRR5278805
ADRDL-011	6,7-nonmotile	1993	Ratite intestine	SAMN06333488	SRR5278804
ADRDL-012	Arechavaleta	1994	Ratite intestine	SAMN06333486	SRR5278803
ADRDL-013	4,5,12:i-monophasic	1994	Ratite liver	SAMN06333485	SRR5380966
ADRDL-014	Berta	1994	Ratite intestine	SAMN06333484	SRR5278808
ADRDL-015	Ituri	1994	Ratite cecum	SAMN06333483	SRR5278773
ADRDL-016	Ituri	1994	Ratite intestine	SAMN06333482	SRR5278772
ADRDL-017	Heidelberg	1993	Wild turkey liver	SAMN06333481	SRR5278779
ADRDL-018	Heidelberg	1993	Wild turkey liver	SAMN06333480	SRR5278777
ADRDL-019	Godesberg	1993	Wild turkey cecum	SAMN06333479	SRR5278778
ADRDL-020	4,5,12:i-monophasic	1993	Eclectus colon	SAMN06333477	SRR5278771
ADRDL-021	Anatum	1993	Giraffe feces	SAMN06333476	SRR5278774
ADRDL-022	Anatum	1993	Giraffe feces	SAMN06333475	SRR5278780
ADRDL-023	Pomona	1993	Python abdominal swab	SAMN06333473	SRR5278767
ADRDL-024	Muenchen	1993	Ratite intestine	SAMN06333472	SRR5278776
ADRDL-025	Typhimurium	1994	Rodent intestine	SAMN06333471	SRR5278770
ADRDL-026	Hadar	1995	Wild chicken intestine	SAMN06333470	SRR5278768
ADRDL-027	Hadar	1994	Ratite intestine	SAMN06333469	SRR5278769
ADRDL-028	Typhimurium	1988	Primate intestine	SAMN06333465	SRR5278873
ADRDL-029	Albany	1988	Saiga intestine	SAMN06333464	SRR5278882
ADRDL-030	Arizona	1988	Snake	SAMN06333462	SRR5330438
ADRDL-031	Arizona	1989	Boa intestinal swab	SAMN06333460	SRR5330446
ADRDL-032	16:z10-e,n,xz15	1989	Cervine feces	SAMN06333459	SRR5330441
ADRDL-033	Enteritidis	1989	Hedgehog spleen	SAMN06333458	SRR5330440
ADRDL-034	Typhimurium(O5−)*	1992	Pigeon airsac swab	SAMN06333457	SRR5330448
ADRDL-035	Typhimurium	1989	Screech owl liver	SAMN06333455	SRR5330445
ADRDL-036	Braenderup	1989	Snow leopard intestine	SAMN06333454	SRR5330444
ADRDL-037	Saintpaul	1989	Snow leopard lung	SAMN06333453	SRR5330406
ADRDL-038	Montevideo	1992	Cervid intestine	SAMN06333451	SRR5329403
ADRDL-039	Enteriditis	1993	Emu feces	SAMN06333450	SRR5329404
ADRDL-040	Enteriditis	1993	Emu feces	SAMN06333449	SRR5380965
ADRDL-041	Worthington	1992	Quail intestine	SAMN06333448	SRR5380958
ADRDL-042	II 43:z4,z23:- or IIIa 43:z4,z23:- or Farmingdale or IV 43:z4,z23:-*	1992	Reptile eggsac	SAMN06333447	SRR5329405
ADRDL-043	Panama	1992	Rhea intestine	SAMN06333694	SRR5409894
ADRDL-044	Ituri	1994	Ratite cecum	SAMN06333692	SRR5409893
ADRDL-045	Newport	1995	Ratite feces	SAMN06333691	SRR5409493
ADRDL-046	Newport	1995	Dolphin lung	SAMN06333689	SRR5409890
ADRDL-047	Typhimurium	1997	Psittacine lung	SAMN06333684	SRR5409485
ADRDL-048	Typhimurium	1997	Psittacine intestine	SAMN06333683	SRR5409315
ADRDL-049	Muenchen	1996	Ratite intestine	SAMN06333682	SRR5409313
ADRDL-050	Schwazengrund	1997	Ratite intestine	SAMN06333681	SRR5409312
ADRDL-051	Archavaleta	1997	Antelope intestine	SAMN06333692	SRR5409893
ADRDL-052	Infantis	1997	Fish water	SAMN06645614	SRR5398012
ADRDL-053	Bredeney	1998	Llama intestine	SAMN06333861	SRR5409360
ADRDL-054	Plymouth	1997	Reptile liver	SAMN06330627	SRR5278827
ADRDL-055	Montevideo	1997	Reptile intestine	SAMN06645663	SRR5398013
ADRDL-056	Branderup	1995	Wild chicken intestine	SAMN06645590	SRR5387496
ADRDL-057	Enteriditis	1996	Wild chicken intestine	SAMN06645569	SRR5387492
ADRDL-058	Typhimurium	1996	Wild chicken feces	SAMN06645567	SRR5387491
ADRDL-059	Bredeney	1995	Gamebird intestine	SAMN06645592	SRR5387497
ADRDL-060	Livingston	1996	Gamebird intestine	SAMN06645590	SRR5387496
ADRDL-061	Enteriditis	1995	Psittacine intestine	SAMN06645588	SRR5387490
ADRDL-062	Montevideo	1996	Psittacine liver	SAMN06645587	SRR5387493
ADRDL-063	7,14:K-monophasic	1995	Ratite intestine	SAMN06645585	SRR5387523
ADRDL-064	Anatum	1995	Ratite feces	SAMN06645654	SRR5387521
ADRDL-065	Enteriditis	1995	Ratite	SAMN06645582	SRR5387527
ADRDL-066	Thompson	1995	Ratite cloacal swab	SAMN06645594	SRR5387519
ADRDL-067	Thompson	1995	Ratite cloacal swab	SAMN06645593	SRR5387517
ADRDL-068	4,5,12: i	1995	Ratite pericardial fluid	SAMN06645652	SRR5387518
ADRDL-069	Livingston	1996	Llama intestine	SAMN06645650	SRR5387514
ADRDL-070	Uganda	1999	Cervine intestine	SAMN06645664	SRR5398014
ADRDL-071	Lille	2000	Cervine intestine	SAMN06645663	SRR5398013
ADRDL-072	Parera	1998	Iguana cloacal swab	SAMN06645662	SRR5398016
ADRDL-073	Anatum	1998	Ratite feces	SAMN06645661	SRR5398025
ADRDL-074	Anatum	1998	Ratite feces	SAMN06645615	SRR5398018
ADRDL-075	Kiambu	1998	Ratite cloacal swab	SAMN06645614	SRR5398012
ADRDL-076	Marina	2000	Reptile feces	SAMN06645660	SRR5398017
ADRDL-077	Bredeney	2003	Alpaca liver	SAMN06645613	SRR5398015
ADRDL-078	Sandiego	2003	Alpaca feces	SAMN06645612	SRR5398009
ADRDL-079	Sandiego	2003	Alpaca feces	SAMN06645611	SRR5398010
ADRDL-080	Bredeney	2003	Antelope feces	SAMN06645610	SRR5398011
ADRDL-081	Virginia or Muenchen*	2002	Ratite	SAMN06645609	SRR5398008
ADRDL-082	Newport*	2002	Ratite	SAMN06645659	SRR5398007
ADRDL-083	Enteritidis*	2002	Ratite	SAMN06645658	SRR5398001
ADRDL-084	Oranienburg	2003	Iguana cloacal swab	SAMN06645657	SRR5398006
ADRDL-085	Give	2003	Iguana cloacal swab	SAMN06658957	SRR5409330
ADRDL-086	Chameleon	2003	Iguana cloacal swab	SAMN06333875	SRR5387539
ADRDL-087	Typhimurium	2002	Llama feces	SAMN06333874	SRR5387538
ADRDL-088	Anatum	2003	Llama feces	SAMN06333873	SRR5387533
ADRDL-089	Typhimurium	2003	Llama feces	SAMN06333872	SRR5387534
ADRDL-090	Agona	2003	Marsupial intestine	SAMN06333871	SRR5387532
ADRDL-091	Miami	2001	Reptile fecal swab	SAMN06658960	SRR5409328
ADRDL-092	Arizona	2001	Reptile liver	SAMN06658959	SRR5409327
ADRDL-093		2001	Reptile cloacal swab	SAMN06658958	SRR5409325
ADRDL-094	Marina	2002	Reptile cloacal swab	SAMN06658962	SRR5409322
ADRDL-095	Marina	2002	Reptile abscess swab	SAMN06658961	SRR5409324
ADRDL-096	Arizona	2002	Reptile lung	SAMN06333869	SRR5387526
ADRDL-097	Parera	2002	Reptile cloacal swab	SAMN06333866	SRR5397979
ADRDL-098	Chameleon	2002	Reptile cloacal swab	SAMN06333865	SRR5397978
ADRDL-099	Senftenberg	2002	Reptile cloacal swab	SAMN06333864	SRR5397977
ADRDL-100	Arizona	2002	Reptile cloacal swab	SAMN06333863	SRR5409363
ADRDL-101	Arizona	2002	Reptile cloacal swab	SAMN06333862	SRR5409361
ADRDL-102	Kisarwe	2003	Reptile cloacal swab	SAMN06333861	SRR5409360
ADRDL-103	Newport	2003	Turtle intestine	SAMN06333859	SRR5409359

* Predicted serovar using Seqsero

### Genomic DNA isolation and WGS

Genomic DNA was isolated from 1.0 mL overnight cultures using the Qiagen DNeasy kits (Qiagen, Valencia, CA, USA) according to manufacturer’s protocol. The quality of isolated DNA was analyzed using NanoDrop™ One (Thermo Scientific™, DE) and was quantified using Qubit^®^ 3.0 (Thermo Fisher Scientific Inc., MA) fluorometer and stored at − 20 °C until use. Whole-genome sequencing was performed on Illumina Miseq platform using V2 chemistry with 2 × 250 paired-end chemistry Briefly, the concentrations of genomic DNA samples were adjusted to 0.3 ng/µL concentration and were processed using Nextera XT DNA Sample Prep Kit (Illumina Inc. San Diego, CA). The libraries were normalized using bead-based procedure and pooled together at equal volume. The pooled library was denatured and sequenced using Miseq reagent version 2 (Illumina, Inc., CA).

### Genome assembly and identification of resistance and virulence genes

The raw data files were de-multiplexed and converted to FASTQ files using Casava v.1.8.2. (Illumina, Inc, San Diego, CA). The FASTQ files were trimmed and assembled de novo using CLC Genomics workbench 9.4 (Qiagen Bioinformatics, CA). The antibiotic resistance genes in the assembled *Salmonella* genomes were identified by BLAST search against a local copy of the antibiotic resistance gene sequence data from ResFinder [9] and CARD [10]. The parameters used for BLAST search were ≥ 95% gene identity and 50% sequence length of the resistance gene. The virulence genes in the genomes were predicted using a similar approach. *Salmonella* virulence gene sequences were extracted from Virulence Factor Database [11] and *Salmonella* genome assemblies were searched against these sequences using BLAST with ≥ 90% gene identity and 50% sequence length cut off.

### Serotyping and antimicrobial susceptibility test

Serotypes of the strains were determined at the National Veterinary Service Laboratory, Ames, IA. Antimicrobial susceptibility of all *Salmonella* isolates was determined using the Sensititre NARMS Gram Negative Plate (CMV3AGNF, Thermofisher). The antibiotics used were gentamicin, streptomycin, amoxicillin–clavulanic acid, ampicillin, cefoxitin, ceftiofur, ceftriaxone, azithromycin, chloramphenicol, nalidixic acid, ciprofloxacin, sulfisoxazole, trimethoprim–sulfamethoxazole, and tetracycline. The AMR was determined according to Clinical and Laboratory Standards Institute guidelines except for azithromycin and sulfisoxazole where the data obtained was indeterminate and were not included in further analysis.

## Results and discussion

### Distribution of Salmonella isolates among wildlife and exotic pets

A total of 103 *Salmonella* isolates sampled between 1988 and 2003 from wildlife and exotic pets were included in the present study for determining the antimicrobial susceptibility using whole genome sequencing. Among 103 isolates, 52 isolates (50.48%) were from wild birds, 1 isolate (0.9%) was from fish, 25 isolates each (24.27%) were from reptiles and mammals (Table 1). The serovars of 96 isolates in this study were determined at the National Veterinary Service Laboratory, Ames, IA, and the remaining 6 serovars were predicted using Seqsero [12]. The serovar of one isolate (ADRDL-093) was not identified under Kauffmann-White classification. A total of 45 serovars were identified among the 103 isolates, of which Typhimurium (12.62%) was the most frequent serovar. Other serovars that had higher prevalence were Enteritidis (6.8%), Anatum (5.8%), Arizona (5.8%), Bredeney (3.9%) and Montevideo (3.9%). The presence of multiple serotypes in wildlife has also been reported from previous epidemiological studies. Nine *Salmonella* samples isolated from marine mammals and birds in California yielded 7 serovars [4]. Similar to our findings, *Salmonella* Typhimurium was reportedly the predominant serovar present in wildlife [13–15] in various parts of the world.

### Phenotypic resistance to antimicrobials

**Fig. 1:**
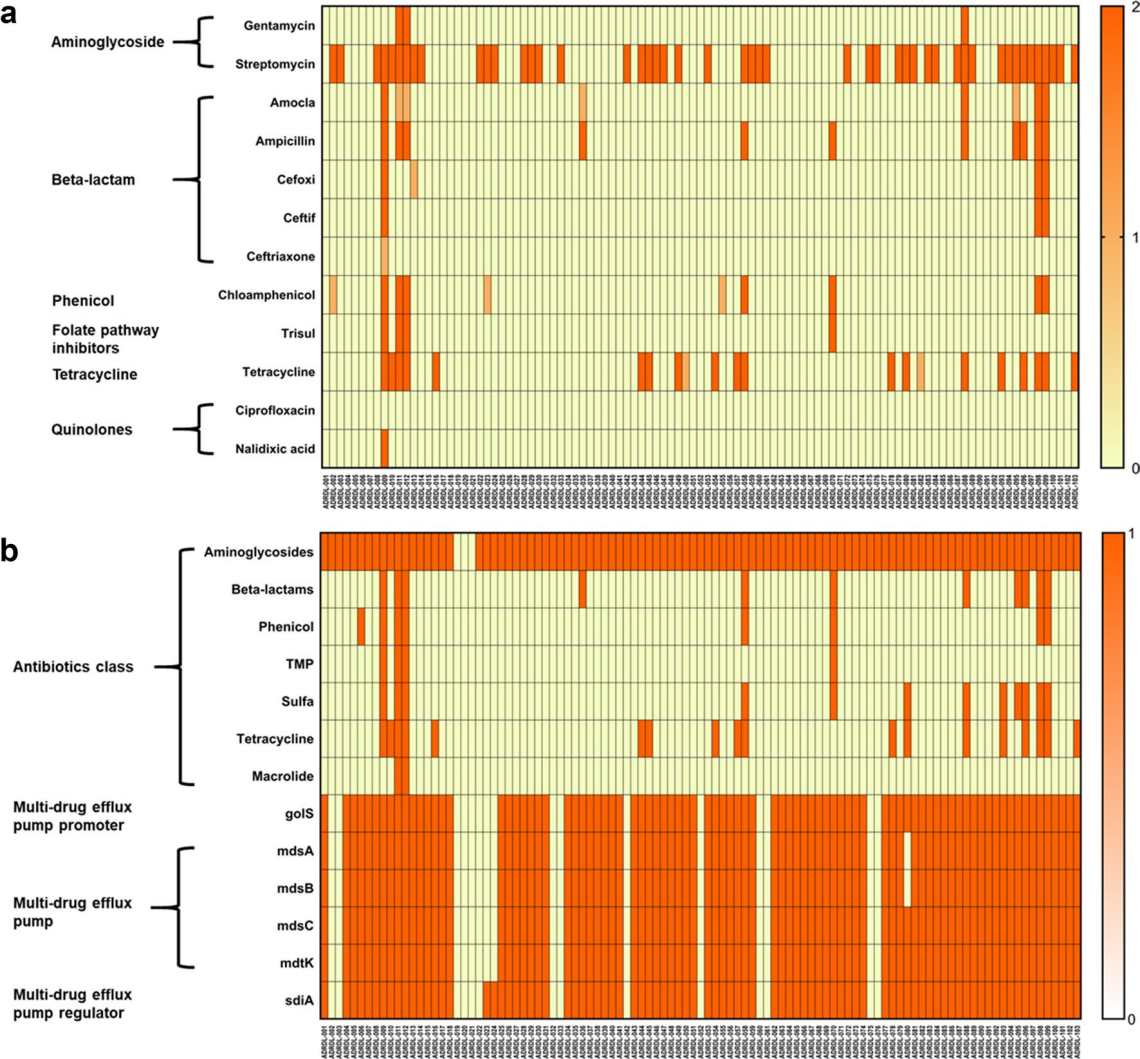
Phenotypic and genotypic anti-microbial resistance of 103 wildlife salmonella isolates. **a** Heatmap of phenotypic resistance against 12 antibiotics was measured using Sensititre NARMS gram-negative plate. The CLSI breakoff points for resistance against various antibiotics was used for determining the antimicrobial susceptibility of 103 salmonella strains. Legend description: 0 = susceptible, 1 = intermediate, and 2 = resistant. **b** Heatmap of genotypic resistance against antimicrobials detected using CLC workbench 9.0 by BLAST against ResFinder 2.1 and CARD database. Legend description: 0 = absent, 1 = present. The golS, mdsABC complex, and mdtK genes associated with multidrug resistance was present in all except 16 isolates. Similarly, multidrug efflux pump regulator gene sdiA was absent in 14 isolates. Complete data used to generate **b** is given in Additional file 2: Table S2

Antimicrobial susceptibility test of 103 *Salmonella* bacterial isolates was performed using Sensititre NARMS gram-negative plate. The results were classified into 3 categories: resistant, intermediate, or susceptible. Fifty-two out of the 103 isolates (50.48%) showed resistance to at least one antibiotic (Fig. 1a). Resistance against the aminoglycoside streptomycin was most commonly observed. Forty-eight of the 103 isolates (46.6%) exhibited this phenotype. However, only three isolates (2.9%) showed resistance to gentamicin which also belonged to the aminoglycoside class of antibiotics. The isolates with resistance against gentamicin were also resistant to streptomycin. In the beta-lactam group, ampicillin resistance was the most common phenotype and was seen in 11 of the isolates (10.67%). Among these 11 isolates, few also shared resistance against other beta-lactams such as amoxicillin–clavulanic acid (4), cefoxitin (3), and ceftiofur (3). All the isolates were susceptible to ceftriaxone except one with intermediate resistance. The isolates that were susceptible to ampicillin were also susceptible to all other beta-lactams. Chloramphenicol resistance was observed for seven isolates (6.7%), trimethoprim–sulfamethoxazole resistance in 4 (3.88%) and tetracycline resistance in 19 (18.44%) of the isolates. All the isolates were susceptible to ciprofloxacin and all except one isolate was susceptible to nalidixic acid. Nine isolates were found to be multi-drug resistant having resistance against more than three antibiotics.

### Genotypic resistance to antimicrobials

The presence of genes that could contribute to AMR was detected by BLAST searching the assembled *Salmonella* genomes against a local copy of Resfinder and CARD sequence data (Fig. 1b). Additional details on the query length and percentage of gene identity for the BLAST results are provided in Additional file 2: Table S2. Bacterial isolates showing “intermediate” resistance on antimicrobial susceptibility test was grouped with “susceptible” isolates for the calculation of sensitivity and specificity of AMR genotype. Twenty-two genes that provided resistance to aminoglycosides were detected and the genes were present in 100 isolates. The sensitivity was 100% and specificity was 5.45% for resistance against aminoglycosides. The low specificity was probably due to the lack of resistance genes being expressed in vitro. Genes responsible for resistance to beta-lactam antibiotics were detected in 11 isolates which were also resistant by antimicrobial susceptibility test. The plasmid-mediated cephalosporinase gene *blaLAT*-1, plasmid-borne class C beta-lactamase gene *blaBIL*-1, and *blaCMY* (Class C) genes were found together and were detected in three isolates. Genes belonging to blaTEM (class A) were found in eight isolates. Collectively, there were 280 beta-lactamase genes present in those 11 isolates. The sensitivity and specificity was 100% for beta-lactams. Phenicol resistance encoded by *cat*, *catA1*, and *floR* genes was present in 8 isolates. The sensitivity was 100% and specificity was 98.96% for phenicol resistance. *dfrA1*, *dfrA10*, *dfrA12*, *sul1*, *sul2*, and *sul3* genes conferring resistance to trimethoprim–sulfamethoxazole drugs were present in 12 isolates. The sensitivity was 100% and specificity was 91.92% for trimethoprim–sulfamethoxazole. The *sul1*, *sul2*, and *sul3* genes could also contribute to resistance against sulfisoxazole. However, a definite conclusion of genotype–phenotype correlation is lacking due to the absence of antimicrobial susceptibility test data that matches the CLSI recommended breakpoint for resistance against sulfisoxazole. Tetracycline resistance encoded by *tet(A)*, *tet(B)*, *tet(C)*, and *tet(D)* genes for tetracycline efflux pumps were detected in 18 samples all of which were also resistant by antimicrobial susceptibility test. The sensitivity was 94.74% and specificity was 100% for tetracycline resistance. Two isolates carried the *mph(A)* gene which confers resistance to macrolides. However, the only macrolide that was tested was azithromycin and the genotype–phenotype relation could not be established due to lack of data from antimicrobial susceptibility test that matches with the breakpoint recommended by CLSI (> 32 mg/L).

Overall, the sensitivity for detecting AMR using genotype was 100% except for tetracycline where 1 isolate was phenotypically resistant even in the absence of the (tet) gene. The specificity for aminoglycosides had the highest degree of incongruence between genotype and phenotype. Fifty-two isolates that were positive for aminoglycoside resistance genes were phenotypically susceptible. Although not to the degree found in this study, a mismatch in phenotype-genotype correlation was also reported previously in *E. coli* and *Salmonella* for aminoglycoside resistance, especially for streptomycin [5, 16]. There was 100% phenotype-genotype correlation for beta-lactam resistance. Phenicols and tetracycline also had > 98% specificity, while trimethoprim–sulfamethoxazole had lower specificity (91.2%) because of four isolates that were genotypically resistant but were phenotypically susceptible. These results are also similar to those obtained in previous studies [5, 16] where correlation approaching 100% was obtained for antimicrobials other than aminoglycosides.

In addition to the genes that confer AMR, we also analyzed the genes that could confer multi-drug resistance (Fig. 1b). The *golS* gene is a promoter for multidrug efflux pump, mdsABC [17] and was detected among 84.46% (n = 87) isolates. Similarly, mdsABC (multidrug transporter of *Salmonella*) complex which is made up of mdsA, mdsB, and mdsC units, was found in all isolates that had *golS* gene except one isolate which lacked *mdsB* and *mdsC* genes. The mdsABC complex is known to provide resistance against a variety of drugs and toxins and is involved in Salmonella virulence and pathogenicity [17, 18]. The *mdtK* gene, a multi-efflux pump which could provide resistance against norfloxacin, doxorubicin and acriflavin [18] and *sdiA*, a regulator for multi-drug resistance pump AcraB [19], were present in 84.46 and 86.41% of the isolates respectively. The presence of these genes could contribute to the virulence and pathogenicity of these *Salmonella* isolates and also indicates the potential for these isolates to resist various antibiotics and toxins.

### Analysis of virulence determinants

**Fig. 2:**
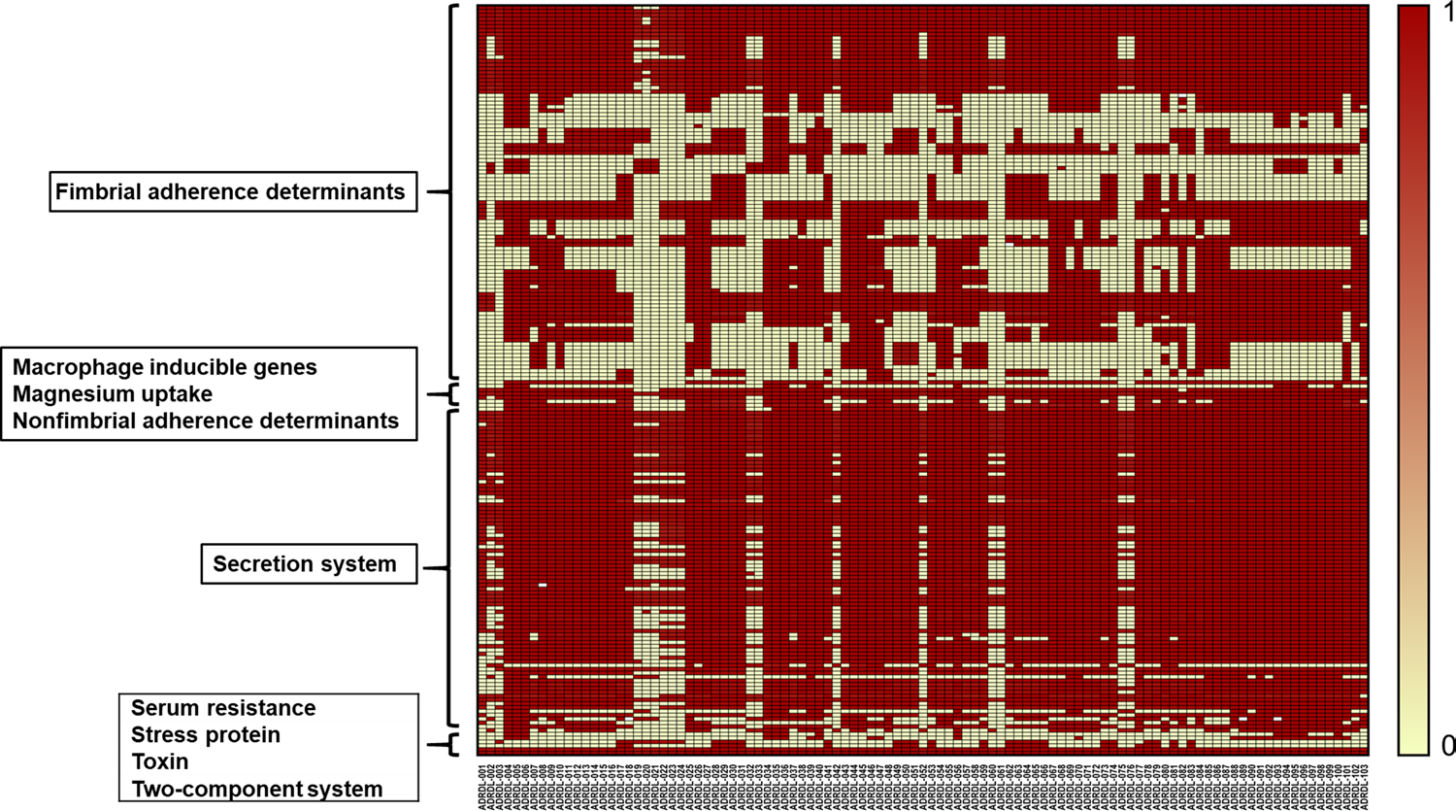
Heatmap of virulence genes present in 103 wildlife salmonella isolates. Salmonella genome Assemblies were searched against a local copy of the Virulence Factor Database using BLAST. In the figure, each row represents a virulence gene and each column denotes a sample. Legend on the left side of the figure denotes the following categories of virulence genes: (I) Fimbrial adherence determinants, (II) Macrophage inducible genes, (III) Magnesium uptake, (IV) Non-fimbrial adherence determinants, (V) Secretion system, (VI) Serum resistance, (VII) Stress protein, (VIII) Toxin, and (IX) Two-component system. The virulence genes belonged 5 categories—PSLT, SeHA, SEN, SeSA, and STM. Seventeen isolates had fewer virulence genes compared to others and this correlated with the absence of genes associated with multidrug resistance. Legend description: 0 = absent, 1 = present. Data underlying this figure is given in Additional file 3: Table S3

The genes that are associated with virulence among 103 wildlife *Salmonella* isolates were analyzed (Fig. 2) using CLC workbench 9.4. The parameters used were the minimum identity of 90% and minimum length of 50%. Additional details on the query length and percentage of gene identity for the BLAST results are given in Additional file 3: Table S3. A total of 197 virulence genes were detected by BLAST search against a local copy of the Virulence Factor Database. The virulence-associated determinants collectively were grouped under 9 categories: fimbrial adherence determinants, macrophage inducible genes, determinants associated with magnesium uptake, nonfimbrial adherence determinants, genes associated with secretion system, serum resistance determinants, stress proteins, toxins, and two-component regulatory systems.

Among fimbrial adherence determinants, the genes belonging to two csg operons *csgBAC* and *csgDEFG* were present universally in all isolates. These genes encode for curli fimbriae or thin aggregative fimbriae and mediate binding to various serum and tissues matrix proteins [20]. Another gene cluster that was ubiquitously present were the *fim* genes that encodes for type 1 fimbriae. This cluster is comprised of the *fimAICDHF* operon and three regulatory genes *fimW, fimY,* and *fimZ* and mediates adherence to eukaryotic cells [21]. However, the *fimY* gene was not detected in ten isolates at the BLAST search cut-off level we used.

The genes belonging to type III secretion system (TTSS/T3SS) encoded by Salmonella pathogenicity island-1 (SPI-1) and -2 (SPI-2) were also predominantly present among the isolates. This included SPI-1 regulator genes *hilACD*, and SPI-1 encoded *inv/spa*, and *prg/org* operons that were detected in all the isolates. Similarly, SPI-2 regulatory gene *ssrB*, chaperone protein-encoding genes—*sscA* and *sscB*, and *ssa* genes that encode for T3SS2 apparatus were also present among 103 isolates. However, the *sse* genes which encode for the effectors were observed only in fewer isolates. Another set of genes that were present in all isolates were the genes that respond to magnesium level in the extracellular environment [22]. This included *mgtc,* which mediates magnesium uptake and *phoP*–*phoQ* genes that are regulators of the two-component system.

The least abundant virulence determinants were the *tcf, sta,* and *pef* fimbrial operons and *spv* gene cluster. These genes belonging to the fimbrial adherence determinants category were detected in less than 25% of the isolates. Additionally, *rck* gene that provides protection against the complement-mediated immune response of the host was also found in low abundance. There were 16 isolates that possessed fewer than 50% of the total virulence genes in the database (Fig. 2). These isolates include ADRDL-002, -003, -019, -020, -021, -022, -023, -024, -032, -033, -042, -052, -060, -061, -075, and -076. Importantly, these isolates also had a lower abundance of genes that contributed to multi-drug resistance (Fig. 1). However, these isolates come under various serotypes and were isolated from different host species. Therefore, a common factor responsible for the observed low abundance of virulence genes is not evident. The universal presence of fimbrial genes and the genes encoded by pathogenicity islands 1–2 among the isolates we report here indicates that these isolates could potentially cause disease in humans. Therefore, the genomes we report here could be a valuable reference point for future traceback investigations in instances where wildlife may be considered as a potential source of human Salmonellosis.

## Additional files

**Additional file 1: Table S1.** Details on genome sequencing and assembly parameters. Salmonella genome assemblies were performed using CLC workbench v 9.4. Quality control of the sequencing data and genome assembly metrics (number of contigs, N50 value, and genome coverage) for each genome is listed.

**Additional file 2: Table S2.** Genotypic Antibiotic resistance gene profile of 103 Salmonella isolates. Salmonella genome assemblies were searched against a local copy of the Resfinder database using BLAST. Cut off parameter for BLAST search was ≥ 95% gene identity and 50% sequence length of the resistance gene. Values in the sample columns indicate the BLAST sequence percentage identity cutoff values.

**Additional file 3: Table S3.** Mapping of virulence genes present in 103 wildlife salmonella isolates. Salmonella genome assemblies were searched against a local copy of the Virulence Factor Database using BLAST. Minimum identity of 90% and minimum length of 50% BLAST hits were used as cut off value. Reference column indicates the NCBI gene locus tag of the reference genes used. Values in the sample columns indicate the BLAST sequence percentage identity cutoff values.

## Abbreviations

AMR: antimicrobial resistance
NARMS: The National Antimicrobial Resistance Monitoring System
WGS: whole genome sequencing
